# The optimized Maxent model reveals the pattern of distribution and changes in the suitable cultivation areas for *Reaumuria songarica* being driven by climate change

**DOI:** 10.1002/ece3.70015

**Published:** 2024-07-17

**Authors:** Xinyou Wang, Zhengsheng Li, Lijun Zhang, Yanlong Wang, Ying Liu, Yushou Ma

**Affiliations:** ^1^ Qinghai Academy of Animal and Veterinary Sciences, Qinghai Provincial Key Laboratory of Adaptive Management on Alpine Grassland, Key Laboratory of Superior Forage Germplasm in the Qinghai‐Tibetan Plateau, Qinghai University Xining Qinghai China

**Keywords:** climate change, ecological niche modeling, Maxent, *Reaumuria songarica*, suitable cultivation areas

## Abstract

*Reaumuria songarica*, a drought‐resistant shrub, is widely distributed and plays a crucial role in the northern deserts of China. It is a key species for desert rehabilitation and afforestation efforts. Using the Maxent model to predict suitable planting areas for *R. songarica* is an important strategy for combating desertification. With 184 occurrence points of *R. songarica* and 13 environmental variables, the optimized Maxent model has identified the main limiting factors for its distribution. Distribution patterns and variation trends of *R. songarica* were projected for current and future climates (2030s, 2050s, 2070s, and 2090s) and different scenarios (ssp_126, ssp_370, and ssp_585). Results show that setting parameters to RM (regulation multiplier) = 4 and FC (feature combination) = LQHPT yields a model with good accuracy and high reliability. Currently, *R. songarica* is primarily suitable for desert control in eight provinces and autonomous regions, including Inner Mongolia, Xinjiang, Qinghai, and Ningxia. The total suitable planting area is 148.80 × 10^4^ km^2^, representing 15.45% of China's land area. Precipitation (Precipitation of the wettest month, Precipitation of the warmest quarter, and Annual precipitation) and Ultraviolet‐B seasonality are the primary environmental factors limiting the growth and distribution of *R. songarica*. Mean temperature of the warmest quarter is the primary factor driving changes in the distribution of suitable areas for *R. songarica* under future climate scenarios. In future climate scenarios, the suitable planting area of *R. songarica* will shrink, and the distribution center will shift towards higher latitude, potentially indicate further desertification. The area of highly suitable habitat has increased, while moderately and less suitable habitat areas have decreased. Increased precipitation within *R. songarica*'s water tolerance range is favorable for its growth and reproduction. With changes in the suitable cultivation area for *R. songarica*, priority should be given to exploring and utilizing its germplasm resources. Introduction and cultivation can be conducted in expanding regions, while scientifically effective measures should be implemented to protect germplasm resources in contracting regions. The findings of this study provide a theoretical basis for addressing desertification resulting from climate change and offer practical insights for the development, utilization, introduction, and cultivation of *R. songarica* germplasm resources.

## Graphical abstract

This study predicted the suitable planting areas of *Reaumuria songarica* in China and the changing patterns of these areas with climate change, providing theoretical support for the future selection of *Reaumuria songarica* for desertification control.

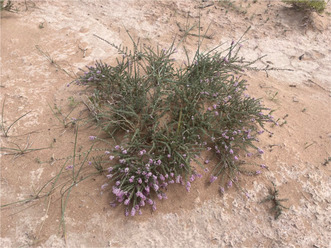

## 1 INTRODUCTION

Climate profoundly influences plant distribution (Yang et al., 2022). Plants inherently inhabit specific ecological niche, adapting their ranges in response to climate fluctuations (Coristine & Kerr, 2015; Menendez et al., 2006; Parmesan & Yohe, 2003). The decline in global biodiversity is partly attributed to climate‐induced alterations in plant habitats and ecosystems (Dong et al., 2023). China has experienced rapid warming compared to the global average over the past century (Shi et al., 2023). Both the Sixth Assessment Report of the Intergovernmental Panel on Climate Change (IPCC AR6) and the World Meteorological Organization (WMO) reported a notable rise in global average surface temperature and precipitation in 2020, ranking it among the three warmest years on record relative to 1850–1900 (Ipcc, 2021; WMO, 2021). Given global warming trends, understanding the distribution patterns and trends of potential plant habitats across diverse climatic is crucial for informing future strategies related to introduction, domestication, ecological restoration, and germplasm resource management (Wei et al., 2018).

Ecological niche models (ENMs) or species distribution models (SDMs) (Brown, 2014) have garnered considerable attention from researchers in recent years (Qin et al., 2020; Zhang et al., 2019). They provide a means to study species distribution, simulate their geographic ranges, and predict their response to climate change. These models can incorporate key elements necessary for species survival based on environmental variables and known distribution data (Sun et al., 2020). Several SDMs such as BioClim, DoMain, GARP, GLM, and Maxent, etc., have been developed based on variations in algorithms (Busby, 1991; Carpenter et al., 1993; Elith et al., 2006; Phillips et al., 2006; Stockwell & Peters, 1999; Yang et al., 2022). The Maxent model developed by Phillips based on the principle of maximum entropy appeared earlier (Phillips et al., 2006). The model, easy to use and quickly to process, provides reliable results, can handle small sample sizes and incomplete species data, while maintaining high stability and accuracy. These advantages have gradually popularized it, making it the most commonly used model in recent years (Sun et al., 2020). Additionally, the predictive outcomes of the Maxent model can be clearly and intuitively visualized using ArcGIS (Geographic Information System), thus enhancing the model's usability (Moreno et al., 2011). Thus far, the Maxent model has successfully assessed the impacts of global climate change on species distribution (Mas et al., 2023; Shi et al., 2023). Additionally, it has guided the introduction and cultivation of ecological and economic tree species (Gong et al., 2022; Tafesse et al., 2023; Zhao, Deng, et al., 2021), addressed limitations of species interactions, aided in establishing and managing protected areas for endangered species (Dias et al., 2023; Liu et al., 2018), and simulated potential invasion ranges of alien invasive species (Lemke et al., 2011; Padalia et al., 2014). Employing the Maxent model to predict suitable areas for ecological restoration tree species and assess their potential planting areas can provide a more scientific and effective guide to restoration ecologically fragmented areas.

**FIGURE 1:**
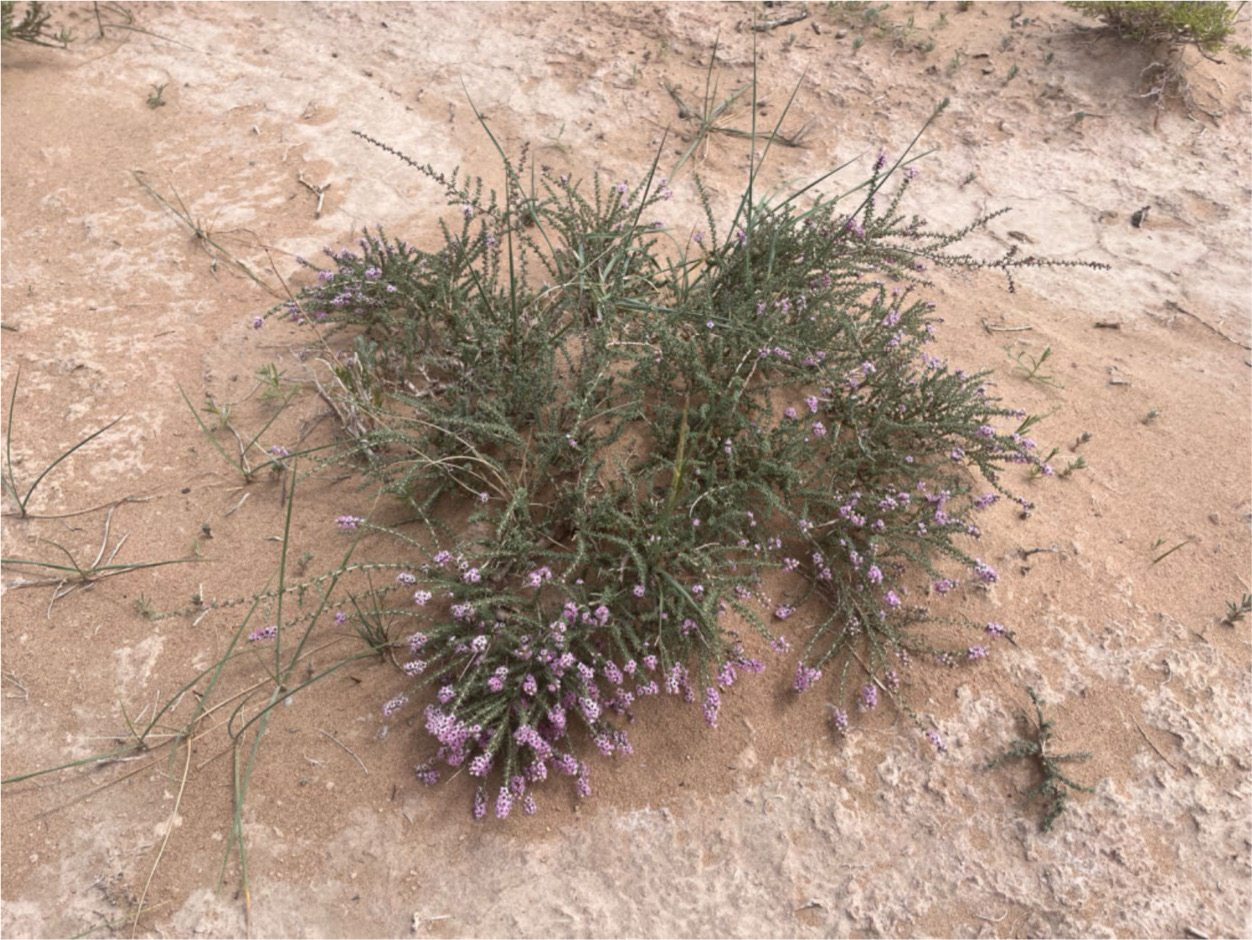
Morphology and habitat map of *Reaumuria songarica*. From Dulan County, Haixi Mongolian and Tibetan Autonomous Prefecture, Qinghai Province, China (97° 54′ 42″E, 36° 39′ 47″N), photograph by Xinyou Wang.

*Reaumuria songarica* is a small, ultra‐arid shrub of the genus *Reaumuria* belonging to the *Tamaricaceae* family (Figure 1) (Lin et al., 1985; Liu et al., 1996). It predominantly occurs in the five northwest provinces of China: Xinjiang, Qinghai, Gansu, Ningxia, and Inner Mongolia. This shrub has well‐developed roots, fleshy leaves (Lin et al., 1985), strong water retention capacity, and low transpiration intensity (Liu et al., 1982; Liu, Zhang, et al., 2007). These characteristics confer a high degree of ecological adaptability to extremely harsh habitats, making it suitable for ecological restoration. Its extensive root system and succulent leaves enable it to effectively utilize groundwater resources and reduce reliance on natural precipitation, allowing it to thrive in water‐deficient deserts (Zhang et al., 2020). Furthermore, its low canopy and dense leaves provide resistance to wind and sand, playing a crucial role in stabilizing soil and controlling shifting sands in these regions. *R. songarica* is an excellent species for ecological management and restoration in these areas. Predicting the potential suitable planting areas and evaluating the limiting environmental factors of ecological tree species like *R. songarica*, in the context of global climate change, holds significant for environmental protection and the stability of ecological balance in the northern desert regions of China.

Global warming has resulted in uneven precipitation distribution, accelerating drought spread in arid regions worldwide (Gong et al., 2022; Sillmann et al., 2019). From a Chinese perspective, the expanding northern desert area posed a serious threat to ecological security. It is crucial to promptly select tree species with broad ecological adaptability for ecological restoration. *R. songarica* demonstrates remarkable resilience and favorable ecological characteristics, and can adapt to the unique environmental conditions of the region, making it the preferred species for combating desertification. This study utilizes the improved Maxent model from the ENMeval package (Muscarella et al., 2014) to conduct predictive analysis of *R. songarica*'s potential habitat. The aim was to address the following questions: (1) What are the primary environmental factors influencing the distribution of *R. songarica*? (2) Under the current climate conditions, in which desert areas of China is *R. songarica* well‐suited for ecological restoration work? (3) How will the potential planting areas of *R. songarica* be impacted by future climate change trends based on shared socio‐economic pathways? Addressing these questions will not only facilitate the domestication and enhancement of *R. songarica* but also offer novel solutions to China's desertification issue.

## 2 MATERIALS AND METHODS

### 2.1 Collection and screening of sample data

**FIGURE 2:**
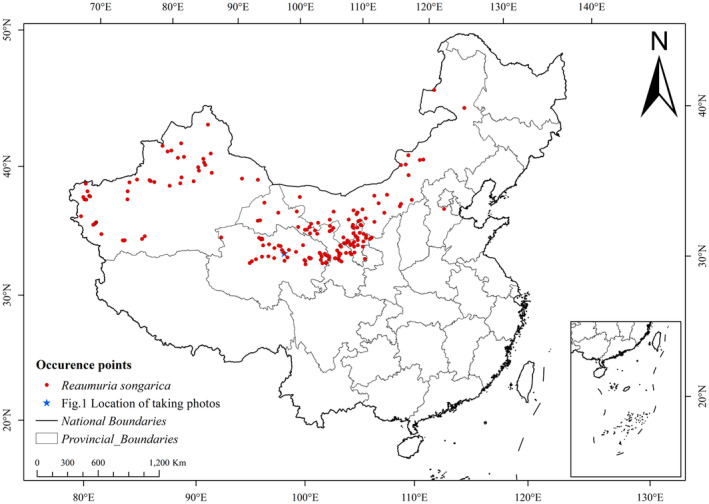
Distributions of occurrence points of *R. songarica*.

We collected 186 distribution records of *R. songarica* in China by searching the Chinese National Plant Specimen Resource Center (https://www.cvh.org.cn), and the Global Biodiversity Information Facility (GBIF) (https://www.gbif.org/). Subsequently, we integrated this data with relevant literature and field surveys, eliminated duplicates ArcGIS 10.8 was utilized to filter the distribution points, ensuring each 10 km × 10 km grid contained only one record. Ultimately, 184 distribution points were obtained for the Maxent model prediction analysis (Figure 2). Specifically, there were 167 distribution sites sourced from the Chinese National Plant Specimen Resource Center and 17 sites from the Global Biodiversity Information Facility.

### 2.2 Variable environment screening and data processing

The initial environmental factors for this study include: 19 bioclimatic variables sourced from the WorldClim database (https://www.worldclim.org/) (Fick & Hijmans, 2017); 17 soil variables obtained from the Harmonized World Soil Database (https://www.fao.org/) (Fischer et al., 2008); three terrain variables acquired from the Geospatial Data Cloud (https://www.gscloud.cn/home); and four radiation variables extracted from the Global Ultraviolet‐B Radiation Dataset (https://www.ufz.de/gluv/) (Beckmann et al., 2014) (Table S1).

We obtained climatic variables for 19 different periods at a 30‐s (1 km × 1 km) resolution from the WorldClim database. The most recent data, released in January 2020, represents the mean for 1970–2000. Future climate data covers the years 2030s (mean of 2021–2040), 2050s (mean of 2041–2060), 2070s (mean of 2061–2080), and 2090s (mean of 2081–2100). Climate data were selected using the representative concentration pathway of the IP6 shared socioeconomic pathway (ssp), specifically ssp_126, ssp_370, and ssp_585, which represent sustainable, localized, and typical development conditions, respectively (Shi et al., 2023). This research employed the Beijing Climate Center Climate System Model 2 Medium Resolution (BCC‐CSM2‐MR). Compared to the BCC‐CSM1.1 model from CMIP5, BCC‐CSM2‐MR offers more accurate estimates for temperature, precipitation, and atmospheric circulation, resulting in more reliable simulation outcomes (Yang et al., 2017). Furthermore, BCC‐CSM2‐MR is the most widely cited global circulation model when the study area is Asia, particularly China (Shi et al., 2023; Yu et al., 2019). Using the Advanced Spaceborne Thermal Emission and Reflection Radiometer Global Digital Elevation Model (ASTER GDEM V3‐30 m), the resolution of soil factors and radiation variables was 30‐s (1 km × 1 km).

The distribution point for *R. songarica* and all environmental factor were initially loaded into Maxent for pre‐modeling to prevent overfitting and reduce accuracy degradation caused by the spatial collinearity of environmental factors (Lan et al., 2022). Pre‐modeling utilized a 25% test set and a 75% training set, with the other parameters set to default. The jackknife test determined the percentage contribution of each environmental variable, while the bootstrapping approach was employed for 10 calculations using 10,000 background points and 500 iterations (Phillips et al., 2006). Subsequently, Spearman correlation analysis were conducted on the environmental factors. Environmental factors with a low percentage contribution and a high correlation (|*r*| ≥ .8) were eliminated (Shi et al., 2023). Ultimately, 13 environmental variables were selected for the Maxent model (Table S2).

### 2.3 Analysis of multivariate environmental similarity surface and most dissimilar variable

Employing current environmental variables as a reference layer for the *R. songarica* fitness zone, the Multivariate Environmental Similarity Surface (MESS) was utilized to assess the scope of climate anomalies under future climate scenarios, and the Most Dissimilar variable (MOD) was analyzed to identify key factors leading to changes in the potential fitness zones of *R. songarica*. Within the reference layer, min_*i*_ and max_*i*_ represent the minimum and maximum values of environmental variable *V*_*i*_, respectively, and *p*_*i*_ denotes the value of environmental variable *V*_*i*_ at point *P* during a specific time period. *f*_*i*_ denotes the percentage of points in the study area where environmental variable *V*_*i*_ is less than *P*_*i*_. When *f*_*i*_ = 0, the MESS is 100 (*P* − min_*i*_)/(max_*i*_ – min_*i*_); when 0 < *f*_*i*_ ≤ 50, the MESS is 2*f*_*i*_; when 50 < *f*_*i*_ < 100, the MESS is 2 (100 − *f*_*i*_); and when *f*_*i*_ = 100, the MESS is 100 (max_*i*_ − 100)/(max_*i*_ – min_*i*_). The MESS for point P represents the minimum similarity value among various environmental variables, referred to as MOD.

MESS and MOD were computed using the “density. Tools. Novel” function within the Maxent software, following the method outlined by Elith et al. A negative MESS value indicates that one or more variables at the point fall outside the reference layer's range, defining it as a climatic anomaly. An MESS value of 100 indicates a completely normal climate, while MOD represents the variable with the highest degree of abnormality, corresponding to the minimum value of relative similarity among the variables. This variable could be a crucial factor influencing the changing distribution of *R. songarica*'s habitat.

### 2.4 Model optimization and accuracy evaluation

Studies have shown that the unoptimized Maxent model is insufficient for accurately predicting species distribution (Halvorsen et al., 2015; Hijmans, 2012; Veloz, 2010; Warren & Seifert, 2011). Two parameters, the regulation multiplier (RM) and feature combination (FC), significantly influence the model's prediction accuracy. Optimizing these parameters can reduce overfitting and improve prediction accuracy.

In this study, we used the R ENMeval package to optimize the FC and RM parameters of the Maxent model (Chala et al., 2016; Merow et al., 2014; Muscarella et al., 2014). Relevant studies indicate that the RM is generally higher than the default value of 1, with models performing best when RM values range between 2 and 4 (Moreno‐Amat et al., 2015; Muscarella et al., 2014; Radosavljevic & Anderson, 2013; Shcheglovitova & Anderson, 2013). Therefore, we selected the following FCs for evaluation: L (linear), LQ (linear and quadratic), H (hinge), LQH, LQHP, and LQHPT. Additionally, we incrementally increased the RM by 0.5 within the range of 0.5–4, resulting in 48 parameter combinations. The ENMeval package was used to comprehensively assess these combinations. The parameter combination with the smallest delta AICc was selected for the final modeling predictions (Lai et al., 2022). The optimal simulation outcomes had a delta AICc value of 0. For this study, we selected LQHPT as the FC with an RM of 4.

After removing the collinearity between the environmental factors, the 13 environmental variables and the spatial point data of *R. songarica* were added to the Maxent model. The optimal combinations of FC and RM were selected, while other variables remained consistent during pre‐modeling. A receiver operating characteristic (ROC) curve was constructed to assess the model's fit to the data.

The quality of the model was evaluated using the area under the ROC curve (AUC). The AUC is a common metric for assessing model accuracy, independent of the model's critical values (Wan et al., 2021; Zhu et al., 2017). Higher AUC values indicate better model fit, with AUC ranging from 0 to 1. Specifically, AUC values less than 0.7 represent poor prediction performance; values between 0.7 and 0.8 represents moderate predictive performance; values between 0.8 and 0.9 represents good predictive performance, and values between 0.9 and 1 represents excellent predictive performance (Warren et al., 2010; Zhao et al., 2022).

We calculated null‐models based on Raes and ter Steege's method to validate the empirical models (Raes & ter Steege, 2007). The results showed that the Maxent model significantly deviated from the null‐model (*p* < .01), indicating an environmental bias in the distribution points of *R. songarica*. Consequently, we constructed a bias corrected null‐model. The results indicated that the Maxent model also significantly deviated from the bias corrected null‐model (*p* < .05). This demonstrates that the Maxent model constructed in this study is superior to the random model, resulting in more credible and accurate outcomes (Figure S1).

The ASC output file from the Maxent model was imported into ArcGIS 10.8 and overlay on the Chinese administrative division map for visualization. Reclassifying the predicted results allowed for the calculation of the corresponding areas. It has been shown that using different ratios of the number of known presences to the number of random points in the Maxent model for the dataset selection of maximum training sensitivity plus specificity Cloglog threshold (MTSPS) produces consistent results. Therefore, in this paper, MTSPS was chosen as a threshold to classify suitable and unsuitable areas (Liu et al., 2016; Zhao, Zhang, et al., 2021). Four categories of suitability were identified: No suitability (*p* < MTSPS, MTSPS value of .3378); Low suitability (.3378 > *p* < .5585); Medium suitability (.5585 > *p* < .7792); and High suitability (0.7792 > *p* < 1).

## 3 RESULTS

### 3.1 Model optimization and accuracy evaluation

The Maxent model was used to predict the potentially planting area of *R. songarica* based on 184 distribution sites of the species and 13 environmental variables. The optimized FC and RM were LQHPT and 4, respectively, compared to the default parameters of LQHP and 1 (with delta. AICc = 0). As shown in Table S3, the optimized Maxent model's AUC value increased from 0.9235 to 0.9414; the delta AICc value decreased from 231.997 to 0; and the mean difference AUC value decreased from 0.0591 to 0.0179. This suggests that the improved parameter combination using the R ENMeval package reduces the complexity and overfitting of the Maxent model, increase the reliability of the forecast results, and produces for the predictions potentially area of *R. songarica*.

Our Maxent model underwent validation by comparing it against a null model. The distribution points of R. songarica exhibited environmental biases, leading to the development of bias‐corrected null models. Results indicated that the Maxent model deviated significantly from these bias‐corrected null models, demonstrating its superior performance over stochastic models and higher accuracy (Figure S1).

### 3.2 Analysis of key environmental variables in potential potentially suitable planting area of *R. songarica*

**FIGURE 3:**
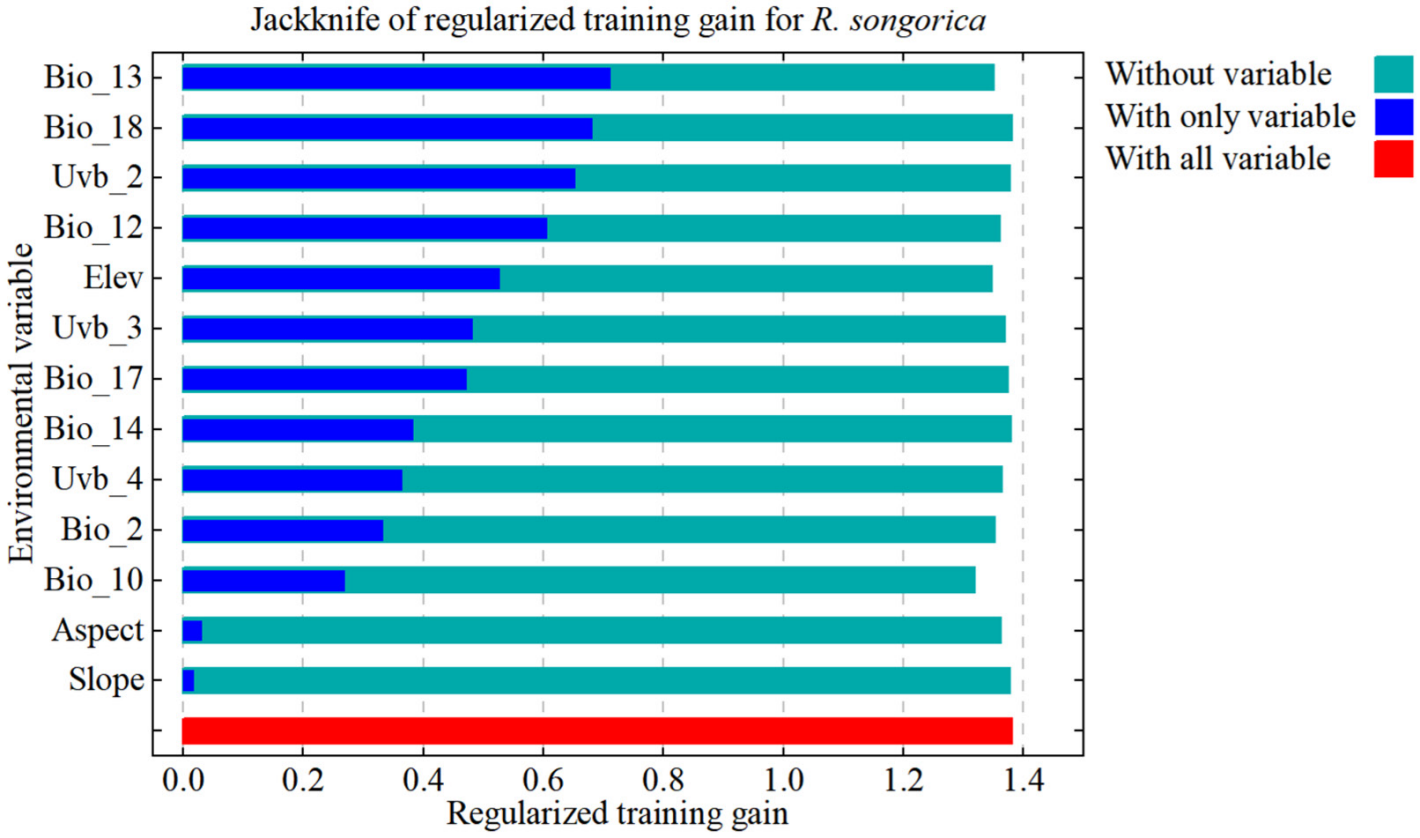
Jackknife test for a single environmental variable.

The jackknife test results for the different environmental variables showed that the regularized training gain was more significant for the precipitation of the wettest month (Bio_13), the precipitation of the warmest quarter (Bio_18), the ultraviolet‐B seasonality (Uvb_2), and the annual precipitation (Bio_12) (Figure 3). These variables had a more substantial influence on the distribution of *R. songarica* compared to other environmental variables, as they contributed more effectively.

In this study, seven out of the 13 environmental factors significantly influenced the distribution of *R. songarica* (Table S4): Precipitation of the wettest month (Bio_13), Mean Ultraviolet‐B of Lowest Month (Uvb_4), Mean temperature of the warmest quarter (Bio_10), Annual precipitation (Bio_12), Altitude, Mean Ultraviolet‐B of Highest Month (Uvb_3), and Ultraviolet‐B Seasonality (Uvb_2). These factors have a cumulative percent contribution of 90.5% and a cumulative permutation importance of 86.9%.

Precipitation is the primary environmental factor affecting the potentially suitable habitat area of *R. songarica* under current climate conditions, followed by ultraviolet‐B and temperature. These findings were derived from the jackknife test results for individual environmental variables and the analysis of their percent contribution and permutation importance. Although the contribution rate of elevation is 5.1% and its permutation importance is 16.6%, elevation primarily influences the distribution of *R. songarica* by altering temperatures and precipitation (Table S4). Other topographical factors, such as slope and aspect, have relatively minor effects on the suitable habitat distribution of *R. songarica*.

In summary, precipitation and ultraviolet radiation remain the primary environmental factors influencing the potential suitable planting area for *R. songarica*, consistent with its biological characteristic as a drought‐tolerant shrub in desert environments.

### 3.3 The suitable planting area of *R. songarica* under current climatic conditions

Based on the optimized Maxent model, the potential cultivation areas for *R. songarica* were visualized using ArcGIS under current climatic conditions. In the figure, red, green, and yellow represent high, medium, and low suitability, respectively, while white indicates unsuitability. According to the predicted results, the suitable habitat of *R. songarica* spans eight provinces and autonomous regions, including Inner Mongolia, Ningxia, and Gansu. In terms of area, it is primarily distributed in Xinjiang (52.50 × 10^4^ km^2^, accounting for 31.01% of the region's area); Inner Mongolia (49.35 × 10^4^ km^2^, accounting for 41.48% of the region's area); Gansu (22.03 × 10^4^ km^2^, accounting for 51.61% of the province's area); Qinghai (17.43 × 10^4^ km^2^, accounting for 24.94% of the province's area); and Ningxia (49.30 × 10^4^ km^2^, accounting for 94.06% of the region's area). In the core areas of the suitable habitat, such as the Loess Plateau and Hetao Plain, the distribution of suitable planting areas is relatively concentrated. However, in peripheral areas, due to the barriers of deserts and basins (such as the Tarim Basin and the Badain Jaran Desert), the distribution of suitable planting areas for *R. songarica* is more scattered and highly fragmented.

Among the 184 distribution points of *R. songarica*, 89 points fell within the highly suitable area according to the forecasted results (48.37%); 49 points fell within the moderately suitable area (26.63%); 24 points fell within the low suitable area (13.04%); and the remaining 22 points were considered unsuitable (11.96%). Most of the 22 points in unsuitable areas are located on the edges of major desert regions (such as the Tarim Basin, Junggar Basin, Qaidam Basin, etc.), like due to the shrinking suitable habitat range of *R. songarica* caused by climate change.

**FIGURE 4:**
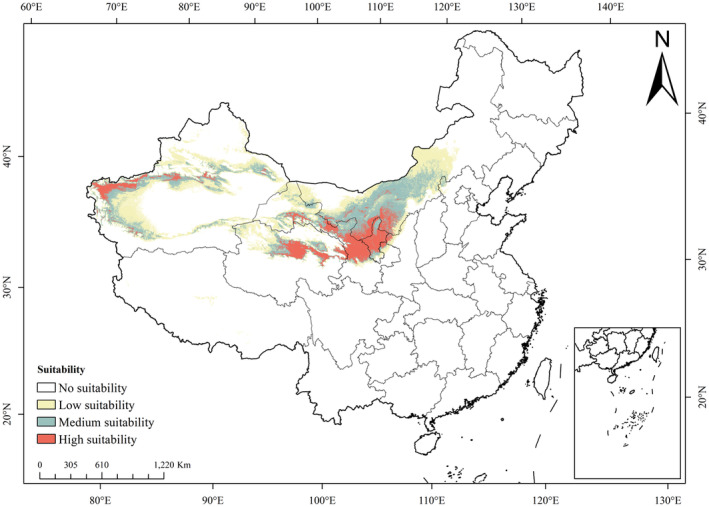
Distribution of suitable areas of *R. songarica* under current climatic conditions.

The high‐suitability zone for *R. songarica* covers 26.56 × 10^4^ km^2^, or 2.76% of the entire study area. This zone includes the western edge of the Qaidam Basin and Hehuang Valley in Qinghai, the western edge of the Tarim Basin in Xinjiang, and the Loess plateau. The medium‐suitability zone spans 51.11 × 10^4^ km^2^ or 5.31% of the study area, predominantly located in the middle and western regions of Inner Mongolia, adjacent to the high‐suitability zone. The low‐suitability zone encompasses 71.13 × 10^4^ km^2^, constituting 7.38% of the study area, primarily in the western part of the Tarim Basin, the Hexi Corridor, and the central portion of Inner Mongolia, with occasionally presence in Tibet. Altogether, these zones total 148.80 × 10^4^ km^2^, or 15.45% of the study area (Figure 4). In conclusion, the suitable habitat of *R. songarica* is mainly distributed in the arid desert areas of northern China and is not prevalent in the relatively well‐watered regions of Northeast China or areas south of the Qinling‐Huaihe Line.

Therefore, *R. songarica* exhibits natural adaptability to the desert areas of northern China, making it a prime candidate for desertification restoration efforts.

### 3.4 Potentially suitable planting area of *R. songarica* under future climate conditions

**FIGURE 5:**
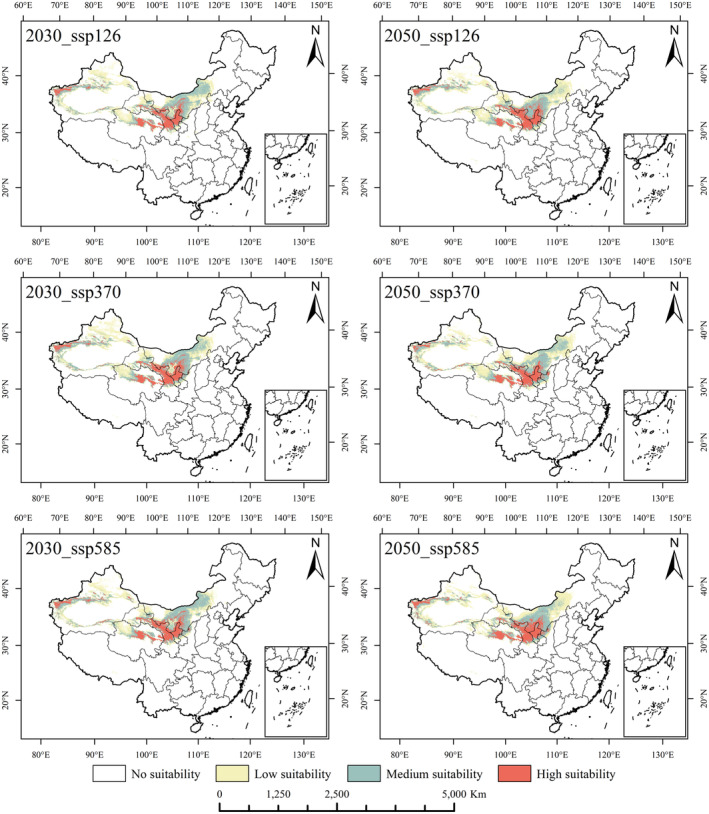
The distribution of potential suitable areas of *R. songarica* under different climate scenarios in 2030s and 2050s.

**FIGURE 6:**
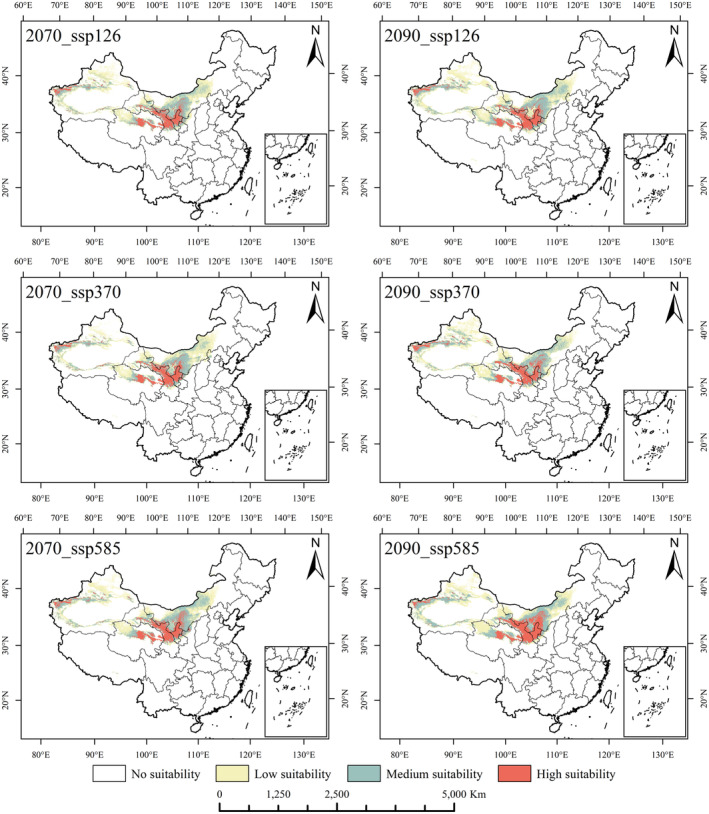
The distribution of potential suitable areas of *R. songarica* under different climate scenarios in 2070s and 2090s.

Using the same methodology and parameters as the predictions under current climatic conditions, we projected the distribution and changes in the prospective suitable zones for *R. songarica* under future climate scenarios. The possible distribution regions of *R. songarica* were determined for periods (2030s, 2050s, 2070s, and 2090s) under three ssps (ssp_126, ssp_370, and ssp_585) (Figures 5 and 6). Analysis of Figures 5 and 6 indicates that the suitable habitat of *R. songarica* undergoes varying degrees of change under future climate scenarios. Geographically, in all three climate scenarios, the high and middle suitability areas for *R. songarica* remain concentrated in the Loess Plateau, Hexi Corridor, Hehuang Valley, Hetao Plain, and along the edges of major desert basins (such as the Qaidam Basin, Tarim Basin, and Junggar Basin). The future potential suitable planting areas for *R. songarica* are relatively consistent with the current distribution characteristics across different climatic scenarios and time periods, with slight variations in the distribution and area of *R. songarica*'s suitable habitat. This suggests that *R. songarica* exhibits strong ecological adaptability to varying degrees of climate change and can effectively contribute to future desert ecological restoration efforts.

Under various future climate scenarios, the suitable cultivation area for *R. songarica* is projected to decrease by 0.12%–13.12% with the exception of the 2023 ssp_126 scenario, which showed an increase of 8.89%. Additionally, the total area of suitable habitat consistently decreased under the ssp_370 scenario across all period. The minimal values for the high, medium, and low suitable areas during this period were 129.28 × 10^4^ km^2^, 24.93 × 10^4^ km^2^, 42.81 × 10^4^ km^2^, and 61.55 × 10^4^ km^2^, respectively. Conversely, the ssp_585 scenario saw expanded of highly suitable habitats across all periods, ranging from 1.61% to 19.39%, peaking at 31.71 × 10^4^ km^2^ in 2090 (Table 1, Figures S2 and S3).

**TABLE 1:** Area and proportion of change in potential suitable areas for *R. songarica* under future climatic conditions.

Period	Climate scenarios	Total suitability		High suitability		Medium suitability		Low suitability	
		Area	Proportion of change	Area	Proportion of change	Area	Proportion of change	Area	Proportion of change
Current	—	148.80	—	26.56	—	51.11	—	71.13	—
2030 s	ssp_126	162.04	8.89%	28.83	8.54%	59.30	16.02%	73.91	3.91%
	ssp_370	143.35	−3.67%	28.03	5.53%	50.49	−1.22%	64.83	−8.86%
	ssp_585	143.03	−3.88%	28.62	7.77%	53.22	4.11%	61.20	−13.96%
2050 s	ssp_126	135.63	−8.85%	27.20	2.42%	45.19	−11.58%	63.24	−11.09%
	ssp_370	139.40	−6.32%	25.64	−3.48%	48.85	−4.43%	64.91	−8.74%
	ssp_585	140.67	−5.46%	30.14	13.46%	44.94	−12.07%	65.60	−7.78%
2070 s	ssp_126	132.78	−10.77%	22.90	−13.77%	44.66	−12.62%	65.22	−8.31%
	ssp_370	129.28	−13.12%	24.93	−6.15%	42.81	−16.25%	61.55	−13.47%
	ssp_585	138.88	−6.67%	26.99	1.61%	48.40	−5.30%	63.49	−10.73%
2090 s	ssp_126	147.45	−0.91%	26.35	−0.78%	49.21	−3.73%	71.89	1.07%
	ssp_370	135.69	−8.81%	26.00	−2.12%	47.02	−8.00%	62.67	−11.89%
	ssp_585	148.63	−0.12%	31.71	19.39%	47.53	−7.01%	69.38	−2.45%

*Note*: The area of potential habitat is expressed in units of × 10^4^ km^2^.

In conclusion, while the overall suitable planting area for *R. songarica* is generally decreasing, there is an expansion in the highly suitable areas and a reduction in moderately to minimally suitable regions. This trend may be a result of exacerbated desertification. In light of this adverse trend, it is imperative to select desert‐dominant species with strong ecological adaptability for desertification control.

### 3.5 Class dynamics of *R. songarica* suitable areas under future climate conditions

**FIGURE 7:**
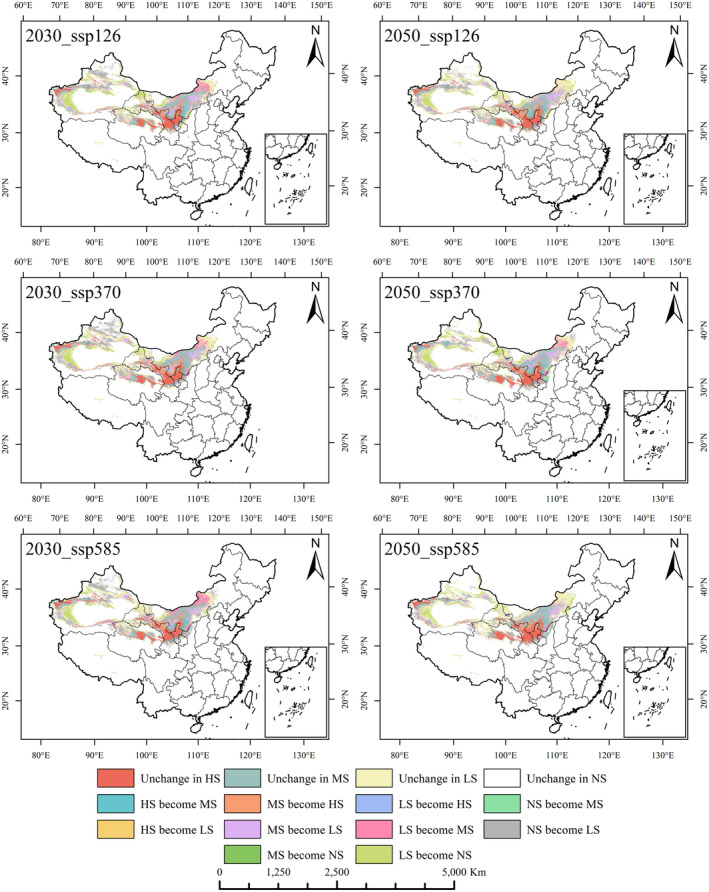
The dynamic change of *R. songarica* suitable area grade under different climate scenarios in 2030s and 2050s. HS represents High suitability, MS represents Medium suitability, LS represents Low suitability, NS represents NO suitability. The same below.

**FIGURE 8:**
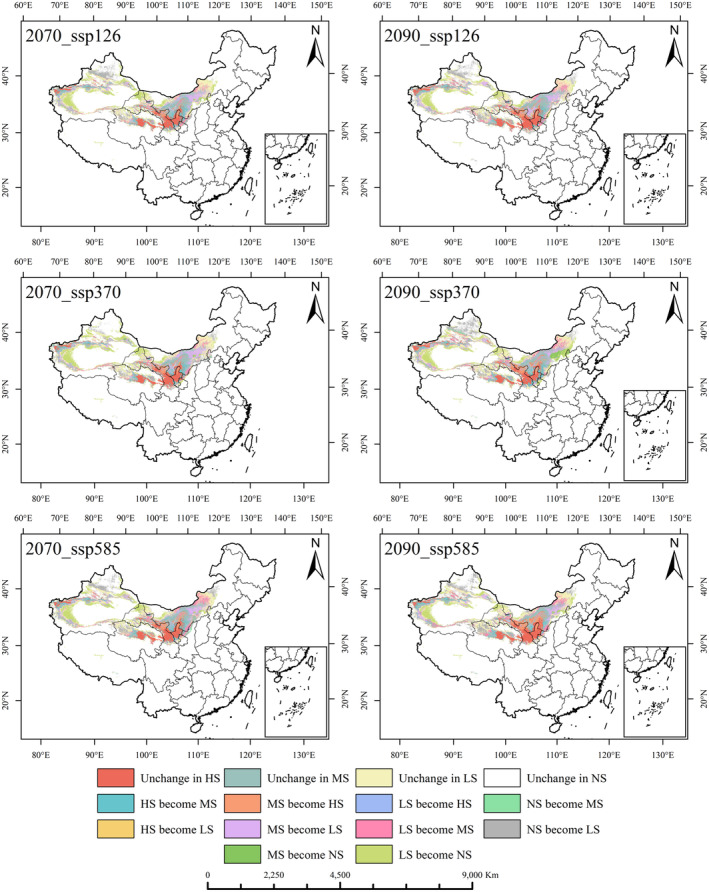
The dynamic change of *R. songarica* suitable area grade under different climate scenarios in 2070s and 2090s.

The analysis, based on the optimized Maxent model, revealed the dynamics of suitable areas for *R. songarica* under different future climate scenarios (Figures 7 and 8). Additionally, ArcGIS 10.8 was employed to compute and analyze the conversion of suitable regions (Table S5).

The Loess Plateau, Hexi Corridor, and HeHuang Valley continuously included in the highly appropriate zone for *R. songarica*. The less suitable zone predominantly lies in the eastern part of the Inner Mongolia Plateau, encompassing the Qaidam Basin and the Tarim Basin region. The moderately suitable zone is primarily located in the Hetao Plain and the northwest periphery of the Tarim Basin. Although varying climate conditions and times periods affect the extent and suitability levels of these zones, most changes occur within the suitable zones themselves. The boundaries of current suitable zones mark the transition from suitable to unsuitable areas. It is anticipated that increased radiation intensity will render some currently low‐suitability zones on the western margin of the Tarim Basin unsuitable, leading to a certain degree of contraction.

The distribution of suitable areas for *R. songarica* exhibits a highly dispersed pattern characterized by high suitability, moderate suitability, low suitability, and unsuitability, in that order. Adjacent levels of suitable undergo more frequent alterations compared to nonadjacent levels. Analysis of Table 1 indicates a projected trend where unsuitable zones for *R. songarica* are like to expand in the future, primarily due to shift from medium and low suitable areas. The largest change in suitable areas involves transitions from low suitable to unsuitability, ranging from 18.78 to 28.67 × 10^4^ km^2^, significantly contributing to the expansion of unsuitable area. The area transitioning between moderate and low suitable areas (MS become LS: 12.06–17.51 × 10^4^ km^2^, LS become MS: 7.43–16.52 × 10^4^ km^2^) exceeds that between high and moderate suitable (HS become MS: 3.31–8.33 × 10^4^ km^2^, MS become HS: 4.59–10.28 × 10^4^ km^2^).

The decreasing suitability of *R. songarica* habitats under changing future climate conditions is evident. Nevertheless, due to its high ecological adaptability to harsh environments, suitable cultivation areas for *R. songarica* remain widely distributed in the northern desert regions of China.

### 3.6 Multivariate environment similarity surface and most dissimilar variable analysis

**FIGURE 9:**
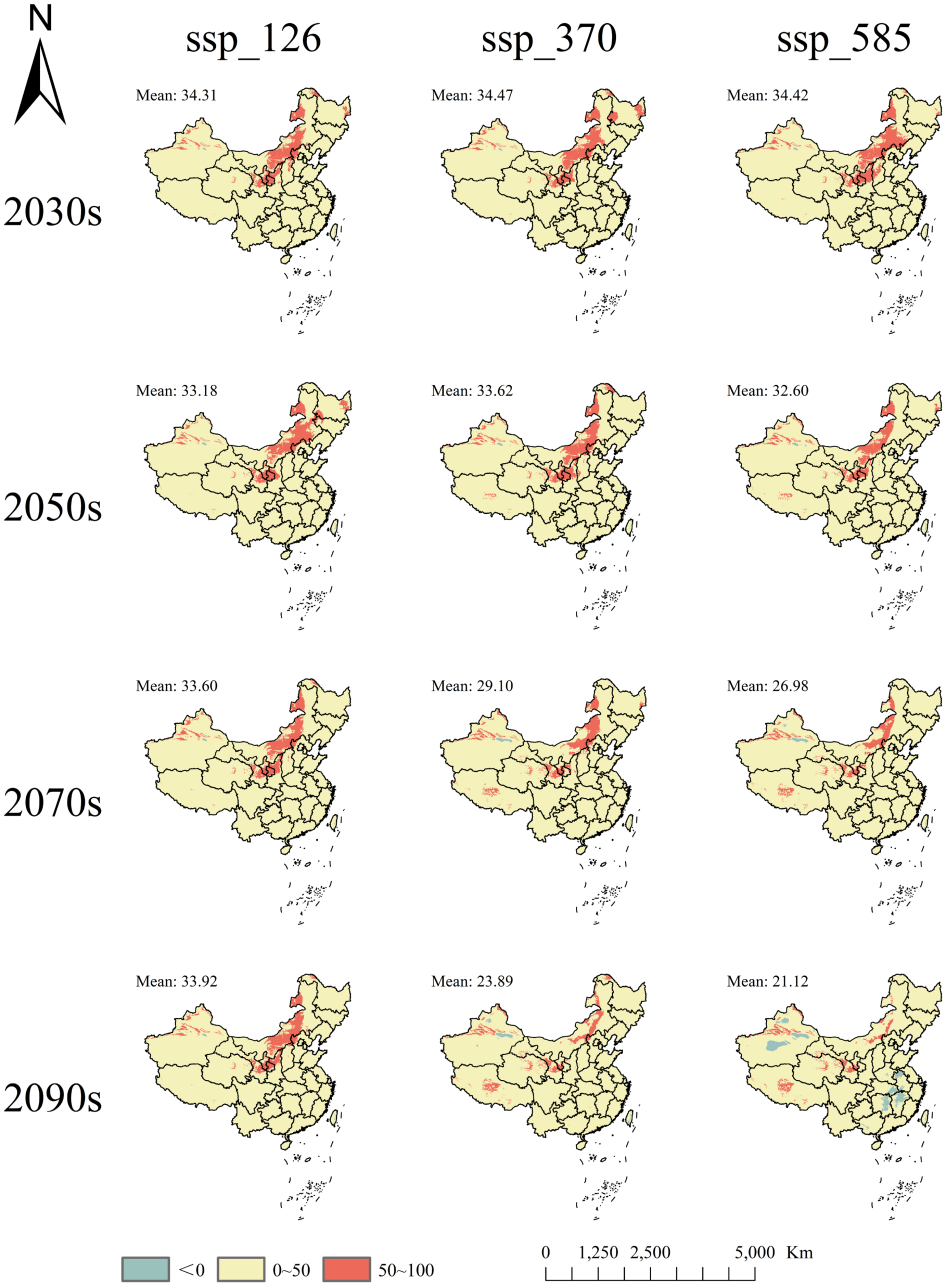
The multivariate environmental similarity surface (MESS) for *R. songarica* under future climate scenarios. The label “Mean: number” in the upper left corner indicates the mean MESS of the 184 *R. songarica* sites under this climate scenario.

**FIGURE 10:**
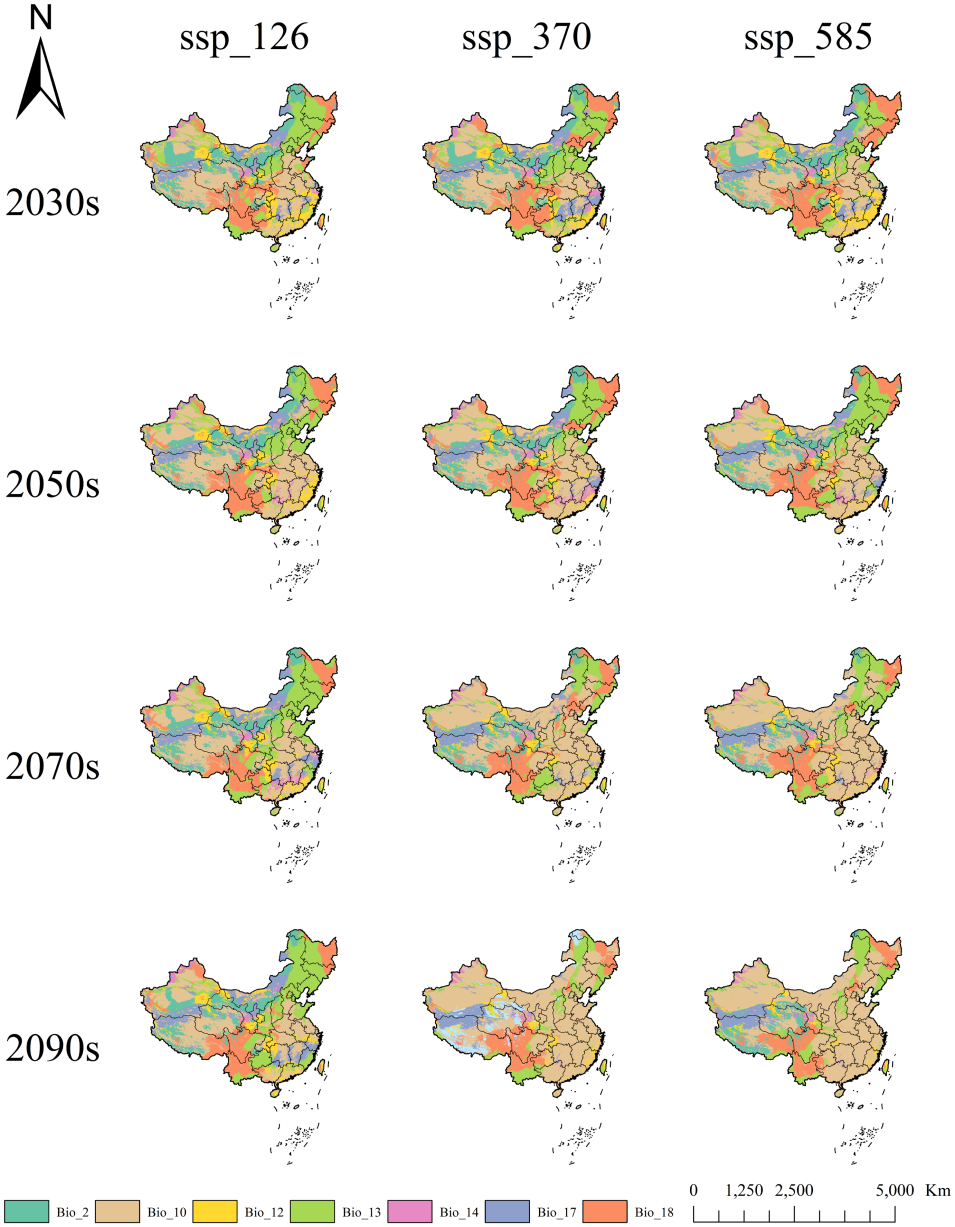
The most dissimilar variable (MOD) for *R. songarica* under future climate scenarios.

Figure 9 shows the mean MESS across 184 distribution points of *R. songarica* under 12 future climate scenarios, ranging from 2023s_ssp126 to 2090s_ssp585, with values ranging from 21.12 to 34.31. Among these scenarios, scenario 2090s_ssp585 was the only one to exhibit 1.63% negative points, indicating the highest degree of climate anomaly, followed by 2090s_ssp370. Across all scenarios, MOD represented the Mean Temperature of the Warmest Quarter, showing that this factor largely influences the highest degree of climate anomaly in *R. songarica* distribution points in future climate scenarios (Figure 10).

### 3.7 Centroid migration in the habitable zone in future climate scenarios

**FIGURE 11:**
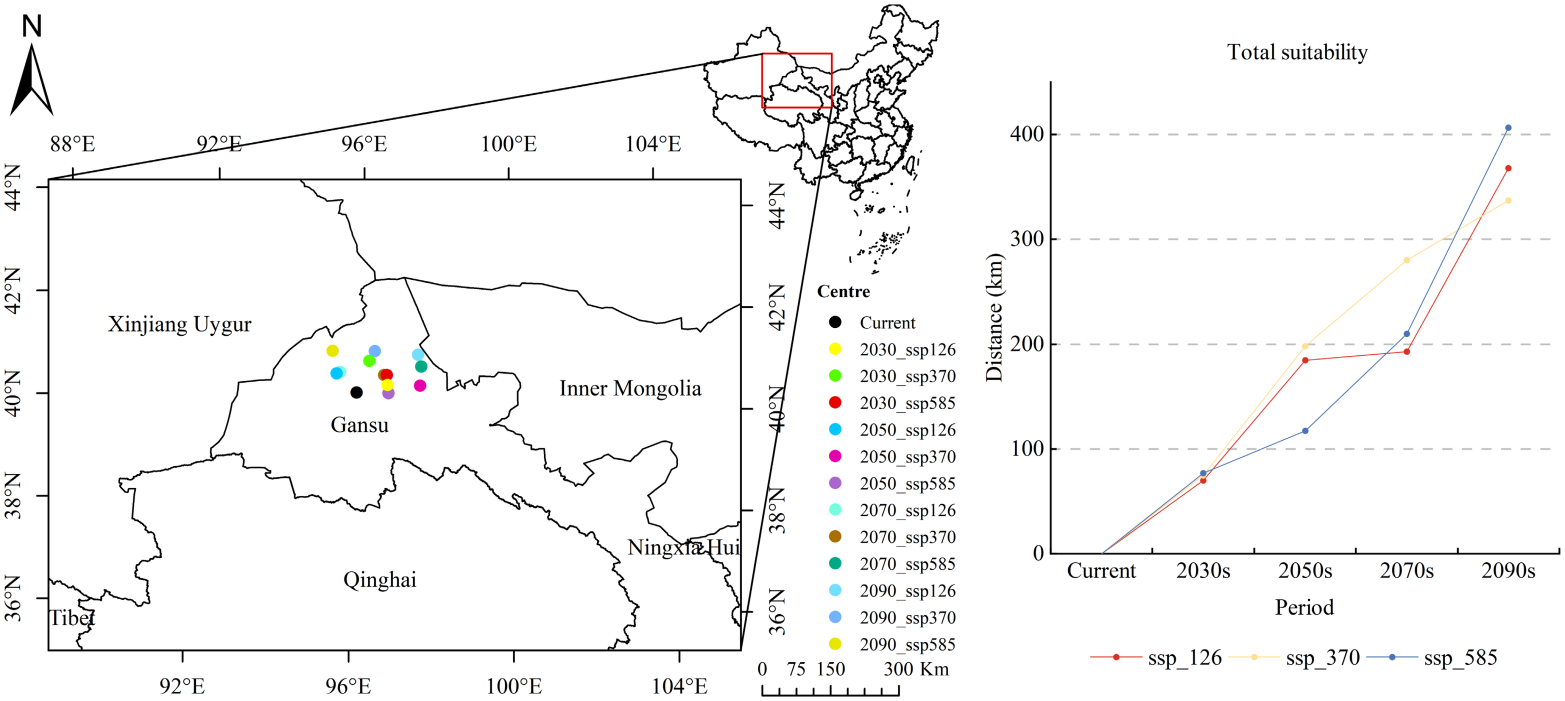
Characteristics of center‐of‐mass distribution and migration distances in suitable areas of *R. songarica* under different time periods and climate scenarios.

**FIGURE 12:**
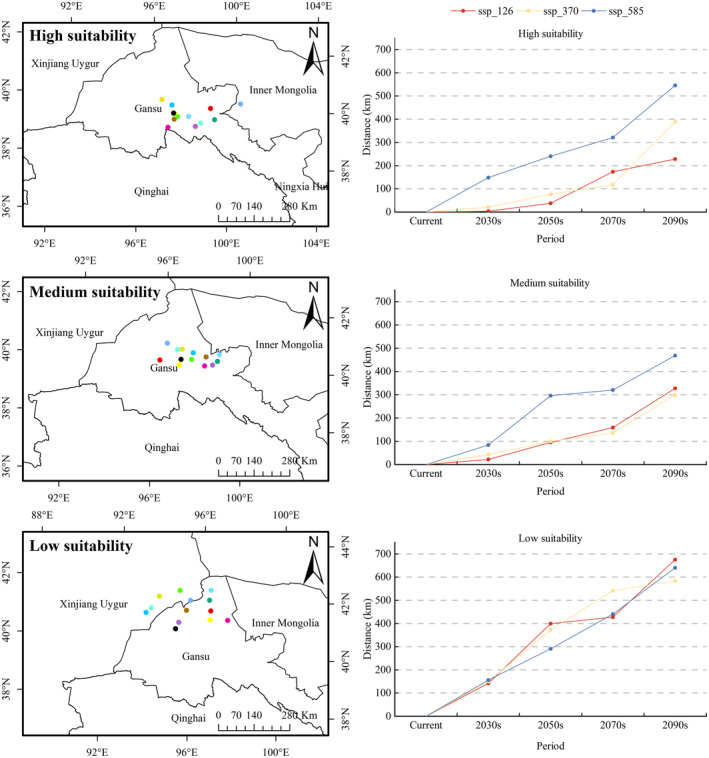
Distribution characteristics and migration distances of the centers of different habitability zones of *R. songarica* under different future climate scenarios. The legend of the distribution of the center of the suitable area in the figure is the same as in Figure 11.

The centroids and migration distances of *R. songarica* suitable regions under present and future climate scenarios were computed using ArcGIS (Figures 11 and 12). Currently, according to the baseline climate scenario, Guazhou County in Gansu Province's hosts the centroid of the *R. songarica* suitable zone (Figure 11). In future climate scenarios, the distribution range of centroids for *R. songarica* suitable areas narrowers, predominantly within Jiuquan City, Gansu Province, encompassing Guazhou County, Subei Mongol Autonomous County, and Yumen County (Figure 12). Overall, centroids tend to shift northward from their current positions (Figure 12). For instance, under the ssp_126 climatic scenario, the centroid of the *R. songarica* suitable zone moves northeast in the 2030s then northwest in the 2050s and 2070s, and returns northeast by the 2090s. Similarly, under the ssp_370 scenarios, future centroids of *R. songarica* suitable areas are located northeast of current centroids. The trend of northward movement persists in the ssp_585 scenario as well. In the 2030s, the centroid of the *R. songarica* suitable zone shifts northeast across all three climate scenarios.

Currently, Yumen City in Gansu Province serves as the focal point of the high suitability zone for *R. songarica*. In future climate scenarios, this high suitability zone shifts southeast within the overall suitability zone. It is widely dispersed across Alashan Right Banner, Jinta County, Yumen City, and nine other counties. Comparing the ssp_585 scenario to the other two climatic scenarios, migration distances from the center point of the high suitability zone were notably greater (Figure 12). Yumen City, located northwest of the center of the high suitability zone, also anchors the current medium suitability zone for *R. songarica* in Gansu Province. Guazhou County, Yumen City, and Jinta County are focal points of the medium suitable area. The ssp_585 scenario exhibits significantly longer migration distances comparable to the other scenarios, similar to those observed from the center point of the high suitability zone (Figure 12). Currently situated in Guazhou County, Gansu Province's, the low fertility zone for *R. songarica* is positioned northwest of the center of the medium fertility zone. Under future climate scenarios, centroids of the low suitability zones for *R. songarica* shift northward to Yizhou District, Guazhou County, and Subei Mongolian Autonomous County. Migration lengths from the center point of the low suitability zone were consistent across the three climate scenarios but notably higher than those from the center points of the high and medium suitability zones (Figure 12).

Based on the above findings, in the future, due to climate change effects on habitats, *R. songarica* is expected to undergo slight migration towards higher latitudes. Furthermore, climate change significantly impacts the high and middle suitability zones for *R. songarica* more than the low suitability zone.

## 4 DISCUSSION

### 4.1 Accuracy of optimized Maxent model and distribution of suitable areas

In this study, the Maxent model was used to forecast probable suitable locations for *R. songarica* under various current and future climate scenarios, based on 184 documented occurrences of *R. songarica* and 13 screened environmental factors.

Phillips tested the default parameters of the Maxent model using a diversity dataset of 226 species from six regions (Phillips & Dudík, 2008). However, adjustments are necessary for specific species research to prevent overfitting and sample bias, which can affect the transferability of species prediction (Hijmans, 2012; Veloz, 2010; Warren & Seifert, 2011). To reduce model overfitting, the FC and RM parameters of Maxent were optimized in this study using the ENMeval program. The AICc of the optimized model decreased from 231.997 to 0, and its AUC increased 0.941 (Table S4), indicating a significant reduction in model complexity and overfitting, thus improving accurate. Xiaodeng Shi demonstrated that the Maxent model's complexity and overfitting were reduced, and accuracy improved, when predicting the possible fitness zones of *Litsea cubeba* with RM = 2.5 and FC = LQH (Shi et al., 2023). Ru Bao found that RM = 4 and FC = LQHPT were the most effective parameters for forecasting the distribution of *Pedicularis longiflora* and *Pedicularis longiflora* var. *tubiformis* fitness zones (Bao et al., 2022). Frederic Sorbe determined that the optimal parameters for analyzing the potential fitness zones of *Ulex europaeus* were RM = 1.5 and FC = H (Sorbe et al., 2023). These findings suggest that optimal parameter combinations for the Maxent model vary among species due to differences in habitat ranges, environmental responses, and biological characteristics.

In this study, empirical models were validated by comparing them against a null model. The results demonstrated that the MaxEnt model developed herein outperformed the null model. Thus, it can be concluded that the distribution of *R. songarica* is largely explained by the MaxEnt model, which operates on the principle of maximum entropy, rather than by a simple null model.

*Reaumuria songarica* is an exceptionally drought‐resistant desert shrub with a robust ability to thrive in extremely arid climates (Liu, Wang, et al., 2007). Based on the estimated distribution area under current climate conditions, *R. songarica* is predominantly found in 8 provinces and autonomous regions across China, including Inner Mongolia, Ningxia, Gansu, Qinghai, and Xinjiang. This finding is consistent with records in “Flora” and early research (Lin et al., 1985; Liu et al., 1982). Geographically, *R. songarica* is primarily concentrated in regions with scant precipitation, such as the Loess Plateau, Hexi Corridor, Qaidam Basin, and Badain Jaran Desert. This distribution is attributed to *R. songarica*'s well‐developed root system and unique biological characteristic of stem splitting reproduction (Liu et al., 1982; Liu, Wang, et al., 2007). According to the predictive results of this study on *R. songarica*'s suitable distribution area, corroborated by previous geographic distribution research (Hao et al., 2014), *R. songarica* emerges as a dominant species in natural desert plant communities (Xia et al., 2018). It often forms natural communities with other drought‐resistant plants like *Nitraria sibirica* and *Tamarix ramosissima*, effectively curbing soil erosion, maintaining ecological system stability, and serving as an excellent species for ecological management (Zheng et al., 2019). With global desertification and extreme weather phenomena on the rise, efforts to combat desertification need intensification (Jiang et al., 2019). Clearly delineating suitable planting areas for *R. songarica* can optimize its ecological traits and maximize its potential in sand control and desertification prevention efforts.

### 4.2 Effects of key environmental variables on the distribution of *R. songarica* suitable cultivation areas

The distribution of suitable areas for *R. songarica* is significantly influenced by several environmental factors, including precipitation of the wettest month, precipitation of the warmest quarter, ultraviolet‐B seasonality, and annual precipitation, as indicated by the Maxent model through percent contribution, permutation importance, and the jackknife test analyses. Among these factors, precipitation emerges as the primary determinant affecting *R. songarica*'s distribution, a finding supported by studies on related species like *R. kaschgarica* and *R. trigyna* (Pahardin et al., 2020). Plants in arid and semi‐arid regions typically exhibit strong drought tolerance, yet water remains a crucial limiting factor for their growth and distribution (D'Odorico & Bhattachan, 2012; Wang et al., 2020). Precipitation influences various physiological processes and metabolic reactions in plants by regulating soil moisture, including carbon fixation (Matthews et al., 2017), transpiration (Koehler et al., 2023), photosynthesis (Oliver et al., 2023), and the synthesis of key metabolites (Herrera, 2009). Consequently, precipitation significantly impacts biomass accumulation, reproduction, and distribution of *R. songarica*. The species demonstrates adaptive strategies such as utilizing shallow soil moisture during periods of ample water and accessing deeper soil layers during dry spells, underscoring the beneficial effects of increased precipitation on its growth and development (Wu et al., 2014). Additionally, the seasonality of ultraviolet‐B radiation profoundly influences *R. songarica*'s distribution. Elevated UV_B levels inhibits plant growth and may reduce seed production, posing challenges to the expansion of desert plant populations (Hidema & Kumagai, 2006; She et al., 2017).

Temperature significantly influences the distribution of suitable habitats for *R. songarica*. While the combined contribution of mean temperature of the warmest quarter and mean daily range during the hottest quarter to *R. songarica*'s habitat suitability was only 11.2%, the mean temperature of the warmest quarter emerged as the most influential variable shaping its distribution under future climate scenarios. Extreme high temperatures may exacerbate drought conditions, potentially limiting *R. songarica*'s ability to survive with its existing water resources, thereby necessitating habitat shifts. We collected distribution data for *R. songarica* across a broad altitude range (24–4522 m). Along with altitude, factors influencing the growth and distribution of *R. songarica* include oxygen content, atmospheric pressure, inter‐species interactions, and bioclimatic variables such as temperature and precipitation. Due to the lack of suitable individual variables representing these factors, elevation was utilized as a composite variable to provide comprehensive information in our model.

### 4.3 Changes in the distribution of *R. songarica* suitable areas in the future

The distribution pattern of plants are expected to undergo significant changes due to climate change (Broennimann et al., 2006; Chen et al., 2011; Parmesan & Yohe, 2003). Future climatic scenarios indicate a shrinking habitat size and a shift in the center of *R. songarica*'s range towards higher latitudes. Recent research suggests that plants typically migrate according to their biological traits in response to temperature and precipitation changes (Chi et al., 2023; Hickling et al., 2010; Shi et al., 2023; Zhao, Zhang, et al., 2021). Drought‐resistant species may migrate towards even drier regions, while cold‐resistant plants may retreat from warmer areas.

As future precipitation is levels are projected to rise, it is expected that the highly suitable habitat area for *R. songarica* will expand, while area classified as moderately and low suitable habitats will likely decrease, consistent with findings for *Litsea cubeba* (Shi et al., 2023). This indicates that increasing precipitation within the plant's tolerance range positively impacts its growth, development, and population expansion. However, habitat degradation has been observed for *R. songarica* in the western Tarim Basin and the southern Badain Jaran Desert, possibly due to desert expansion driven by radiation forcing (Cheng & Shen, 2002), which reduces other critical habitat conditions necessary for the species' survival. This underscores that, under future climate change scenarios, the North China region may face varying degrees of desertification, necessitating enhanced efforts to prevent and control desertification to mitigate ecological damage and its potential threats to human society.

### 4.4 Suggestion for the development and utilization of *R. songarica*

One of today's most pressing ecological and geo‐environmental challenges is desertification. Given China's significant proportion of desertified land, particularly in its northern region (Yang et al., 2023), there is a critical need for vigilant monitoring, prevention, control, and restoration efforts. This study's predictions regarding the suitable habitats of *R. songarica*'s suggest a potential exacerbation of desertification due to escalating global warming, thereby endangering key desert species like *R. songarica*. Urgent and scientifically informed actions are essential to harness these species effectively for sand fixation and desert management, crucial for mitigating desertification processes.

To scientifically and effectively utilize *R. songarica* for desertification control and restoration in northern China, we propose the following recommendations based on our research findings: (1) The primary suitable planting area for *R. songarica* is concentrated in the North China desert region. It is advisable to establish a germplasm resource breeding base in this area, tailored to the local ecological characteristics. (2) In the sandy areas of North China, it is recommended to establish *R. songarica* shelterbelts aimed primarily at windbreak and sand stabilization. This would reduce soil erosion and mitigate the adverse impacts of sandstorms on the ecological environment and human living conditions. (3) Managers should utilize the expansion areas of *R. songarica*'s suitable habitat identified in this study for establishing cultivation sites. In contrast, the contraction areas require increased attention to desertification control and conservation of genetic resources. (4) Desertification control should be conducted without disrupting the original ecological environment. Attention should be given to combining multiple species and the rational use of resources to prevent degradation caused by a single community structure and to avoid further environmental deterioration due to the irrational use of resources.

## 5 CONCLUSION

This study optimized the RM and FC parameters of the Maxent model using the R ENMeval package, thereby reducing the model's complexity and increasing result reliability. The optimized Maxent model was used to analyze the suitable planting areas and climate preference of *R. songarica*. The results indicate that the suitable distribution area of *R. songarica* is concentrated in the northern desert regions of China, such as the Tarim Basin, Qaidam Basin, Loess Plateau, and the Badain Jaran Desert. Precipitation (precipitation of the wettest month, precipitation of the warmest quarter, and annual precipitation) and ultraviolet radiation (UV‐B seasonality) play dominant role in the distribution of *R. songarica*. The most influential variable determining changes in *R. songarica* habitat under future climate scenarios is the mean temperature of the warmest quarter. Under projected future global climate change conditions, the suitable planting area of *R. songarica* will decrease, shifting towards higher latitude. The patterns of changes in suitable habitats areas at various levels suggest that increased precipitation within *R. songarica*'s water tolerance range benefits its growth and reproduction. The northern desert regions of China are expanding, necessitating timely preventive measures to control this trend. This study uses ENMs to precisely assess suitable planting areas and forecast future trends of *R. songarica*, clearly identifying the primary abiotic factors limiting its distribution. This provides a reliable basis for targeted desertification control using such desert‐adapted species.

## AUTHOR CONTRIBUTIONS

**Xinyou Wang:** Conceptualization (lead); data curation (lead); formal analysis (lead); investigation (lead); methodology (lead); software (lead); visualization (lead); writing – original draft (lead); writing – review and editing (lead). **Zhengsheng Li:** Conceptualization (supporting); investigation (supporting); methodology (supporting); supervision (supporting); visualization (supporting). **Lijun Zhang:** Conceptualization (supporting); investigation (supporting); methodology (supporting); software (supporting); supervision (supporting); validation (supporting). **Yanlong Wang:** Investigation (equal); methodology (supporting); software (supporting); supervision (supporting); validation (supporting). **Ying Liu:** Conceptualization (supporting); funding acquisition (equal); validation (equal); writing – review and editing (equal). **Yushou Ma:** Conceptualization (supporting); funding acquisition (lead); investigation (equal); supervision (lead); validation (supporting); writing – review and editing (equal).

## FUNDING INFORMATION

This work was supported by the scientific research project of Chief Scientist Program of Qinghai Province 2024‐SF‐101.

## CONFLICT OF INTEREST STATEMENT

The authors declare that they have no known competing financial interests or personal relationships that could have appeared to influence the work reported in this paper.

## Supporting information


Appendix S1.

## Data Availability

The data that support the findings of this study are openly available in Dryad at https://datadryad.org/stash/share/AxJoT_l2oI8‐JxeZfftGXw7RqiwC_p8IsTd6dCmE_nw; https://doi.org/10.5061/dryad.9p8cz8wqw.
