# Supplementary material for: The optimized Maxent model reveals the pattern of distribution and changes in the suitable cultivation areas for Reaumuria songarica being driven by climate change
Source: Ecol Evol. 2024 Jul 17;14(7):e70015. doi: 10.1002/ece3.70015 (PMC11255383; doi:10.1002/ece3.70015)
Supplement: Supplementary file 1 — Appendix S1. [file ECE3-14-e70015-s001.docx]

**Table S1 Initial environment variable.**

| Type | Code | Description | Unit |
| --- | --- | --- | --- |
| Bioclimatic | Bio_1 | Annual mean temperature | ℃ |
|  | Bio_2 | Mean diurnal range (Mean of monthly (max.temp.-min.temp.) | ℃ |
|  | Bio_3 | Isothermality (bio2/bio7) (×100) |  |
|  | Bio_4 | Temperature seasonality (standard deviation*100) |  |
|  | Bio_5 | Max temperature of the warmest month | ℃ |
|  | Bio_6 | Min temperature of the coldest month | ℃ |
|  | Bio_7 | Temperature annual range (bio5- bio6) | ℃ |
|  | Bio_8 | Mean temperature of the wettest quarter | ℃ |
|  | Bio_9 | Mean temperature of the driest quarter | ℃ |
|  | Bio_10 | Mean temperature of the warmest quarter | ℃ |
|  | Bio_11 | Mean temperature of the coldest quarter | ℃ |
|  | Bio_12 | Annual precipitation | mm |
|  | Bio_13 | Precipitation of the wettest month | mm |
|  | Bio_14 | Precipitation of the driest month | mm |
|  | Bio_15 | Precipitation seasonality (Coefficient of variation) |  |
|  | Bio_16 | Precipitation of the wettest quarter | mm |
|  | Bio_17 | Precipitation of the driest quarter | mm |
|  | Bio_18 | Precipitation of the warmest quarter | mm |
|  | Bio_19 | Precipitation of coldest quarter | mm |
| Soil | T-gravel | Topsoil Gravel Content | %vol. |
|  | T-silt | Topsoil Silt Fraction | % wt. |
|  | T-ref-bulk-density | Topsoil Reference Bulk Density | kg/dm^3^ |
|  | T-oc | Topsoil Organic Carbon | % weight |
|  | T-Ph-H_2_o | Topsoil pH (H_2_O) | -log(H^+^) |
|  | T-esp | Topsoil Sodicity (ESP-exchangeable sodium percentage) | % |
|  | T-ece | Topsoil Salinity | dS/m |
|  | T-cec-soil | Topsoil CEC (soil) | cmol/kg |
|  | Ref-depth | Reference Soil Depth |  |
|  | S-gravel | Subsoil Gravel Content | %vol. |
|  | S-silt | Subsoil Silt Fraction | % wt. |
|  | S-ref-bulk-density | Subsoil Reference Bulk Density | kg/dm^3^ |
|  | S-oc | Subsoil Organic Carbon | % weight |
|  | S-Ph-H_2_o | Subsoil pH (H_2_O) | -log(H^+^) |
|  | S-esp | Subsoil Sodicity (ESP-exchangeable sodium percentage) | % |
|  | S-ece | Subsoil Salinity | dS/m |
|  | S-cec-soil | Subsoil CEC (soil) | cmol/kg |
| Topographical | Elev | Altitude | m |
|  | Slope |  | ° |
|  | Aspect |  | rad |
| Ultraviolet-B | Uvb_1 | Annual Mean Ultraviolet-B | J/m^2^/day |
|  | Uvb_2 | Ultraviolet-B Seasonality | J/m^2^/day |
|  | Uvb_3 | Mean Ultraviolet-B of Highest Month | J/m^2^/day |
|  | Uvb_4 | Mean Ultraviolet-B of Lowest Month | J/m^2^/day |

**Table S2 Environmental variables used in this study.**

| Type | Code | Description | Unit |
| --- | --- | --- | --- |
| Bioclimatic | Bio_2 | Mean diurnal range (Mean of monthly (max.temp.-min.temp.) | ℃ |
|  | Bio_10 | Mean temperature of the warmest quarter | ℃ |
|  | Bio_12 | Annual precipitation | mm |
|  | Bio_13 | Precipitation of the wettest month | mm |
|  | Bio_14 | Precipitation of the driest month | mm |
|  | Bio_17 | Precipitation of the driest quarter | mm |
|  | Bio_18 | Precipitation of the warmest quarter | mm |
| Topographical | Elev | Altitude | m |
|  | Slope | — | ° |
|  | Aspect | — | rad |
| Ultraviolet-B | Uvb_2 | Ultraviolet-B Seasonality | J/m^2^/day |
|  | Uvb_3 | Mean Ultraviolet-B of Highest Month | J/m^2^/day |
|  | Uvb_4 | Mean Ultraviolet-B of Lowest Month | J/m^2^/day |

**Table S3 Evaluation metrics of MaxEnt model before and after optimization**

| Type | FC | RM | AUC | delta. AICc | mean. diff. AUC | mean. OR10 |
| --- | --- | --- | --- | --- | --- | --- |
| Default | LQHP | 1 | 0.9235±0.0164 | 231.997 | 0.0591 | 0.0926 |
| Optimization | LQHPT | 4 | 0.9414±0.0088 | 0 | 0.0179 | 0.0933 |

**Fig.S1 Histogram of frequency distribution of two null-model AUC values**

Note: In the figure AUC C.I. All denotes the AUC value of the random null-model; AUC C.I. Bias denotes the AUC value of the biased corrected null-model.

**Table S4 Analysis of environmental variable percent contributions and permutation importance.**

| Variable | Percent contribution | Permutation importance |
| --- | --- | --- |
| Bio_13 | 46.5 | 14.8 |
| Uvb_4 | 14.6 | 7.9 |
| Bio_10 | 8.3 | 34.2 |
| Bio_12 | 6 | 6.8 |
| Elev | 5.1 | 16.6 |
| Uvb_3 | 5.1 | 6 |
| Uvb_2 | 4.9 | 0.6 |
| Bio_2 | 2.9 | 1.2 |
| Bio_17 | 2.6 | 6.3 |
| Aspect | 1.7 | 1.2 |
| Bio_18 | 1.5 | 0.1 |
| Bio_14 | 0.6 | 3.7 |
| Slope | 0.2 | 0.5 |

**Table S5 The suitable area of each grade is transformed**

| Period  Type | 2030 | | | 2050 | | | 2070 | | | 2090 | | |
| --- | --- | --- | --- | --- | --- | --- | --- | --- | --- | --- | --- | --- |
|  | ssp_126 | ssp_370 | ssp_585 | ssp_126 | ssp_370 | ssp_585 | ssp_126 | ssp_370 | ssp_585 | ssp_126 | ssp_370 | ssp_585 |
| Unchange_HS | 18.62 | 19.89 | 19.08 | 19.86 | 19.10 | 23.06 | 17.76 | 18.56 | 18.84 | 19.95 | 17.29 | 20.39 |
| Unchange_MS | 29.09 | 29.43 | 28.35 | 28.92 | 27.82 | 32.37 | 27.45 | 25.81 | 27.60 | 29.96 | 25.39 | 27.59 |
| Unchange_LS | 31.10 | 33.66 | 35.87 | 37.94 | 34.14 | 43.82 | 34.53 | 33.04 | 32.54 | 38.15 | 31.18 | 37.81 |
| Unchange_NS | 820.09 | 818.81 | 820.10 | 825.98 | 820.75 | 825.21 | 821.93 | 825.24 | 819.13 | 817.54 | 817.28 | 816.49 |
| HS_become_MS | 7.28 | 5.92 | 6.98 | 6.24 | 7.03 | 3.31 | 8.25 | 7.47 | 7.26 | 5.87 | 8.33 | 5.92 |
| MS_become_HS | 5.97 | 7.36 | 8.30 | 6.67 | 5.41 | 6.24 | 4.59 | 5.73 | 7.14 | 5.82 | 7.71 | 10.28 |
| LS_become_HS | 0.22 | 0.48 | 0.74 | 0.35 | 0.72 | 0.50 | 0.15 | 0.35 | 0.67 | 0.28 | 0.73 | 0.87 |
| NS_become_MS | 0.68 | 0.51 | 1.11 | 0.25 | 1.88 | 1.42 | 0.43 | 0.57 | 1.30 | 0.51 | 1.50 | 0.78 |
| HS_become_LS | 0.45 | 0.54 | 0.31 | 0.26 | 0.25 | 0.04 | 0.32 | 0.29 | 0.26 | 0.52 | 0.72 | 0.10 |
| MS_become_LS | 15.06 | 13.48 | 13.73 | 14.64 | 16.81 | 12.06 | 16.78 | 17.51 | 14.65 | 14.78 | 13.05 | 12.34 |
| LS_become_MS | 12.99 | 14.09 | 16.52 | 9.34 | 11.96 | 7.43 | 7.89 | 8.40 | 11.94 | 12.38 | 11.53 | 12.86 |
| NS_become_LS | 15.18 | 16.70 | 20.77 | 9.82 | 13.08 | 9.19 | 12.94 | 10.22 | 15.44 | 17.97 | 17.05 | 18.64 |
| MS_become_NS | 0.77 | 0.62 | 0.89 | 0.72 | 0.93 | 0.29 | 1.79 | 1.70 | 1.46 | 0.38 | 4.70 | 0.82 |
| LS_become_NS | 26.14 | 22.21 | 21.11 | 22.87 | 23.78 | 18.78 | 27.02 | 28.67 | 25.38 | 19.76 | 27.05 | 19.11 |

Note: The area of suitable area changes is expressed in units of × 10^4^ km^2^.


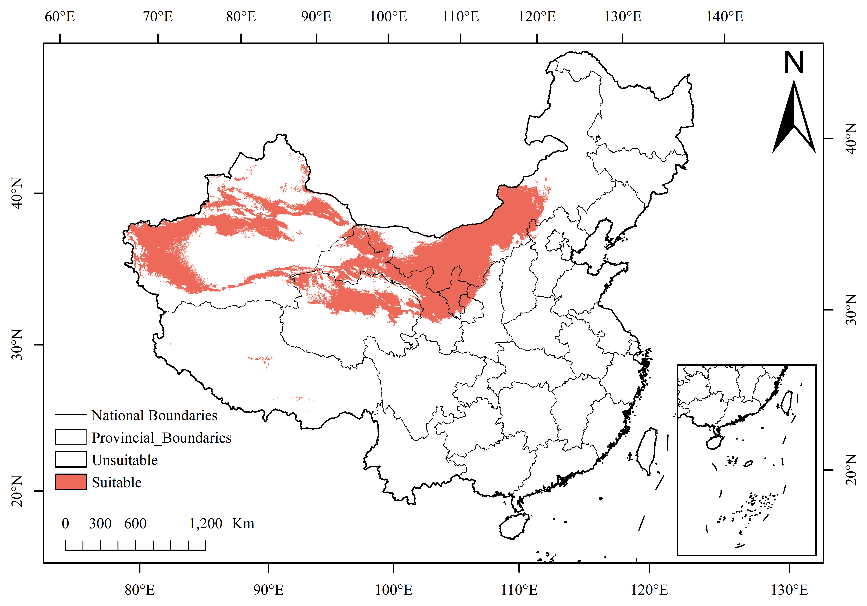


**Fig.S2 Binary map of the distribution of Reaumuria songarica in the current climate (Suitable/Unsuitable)**


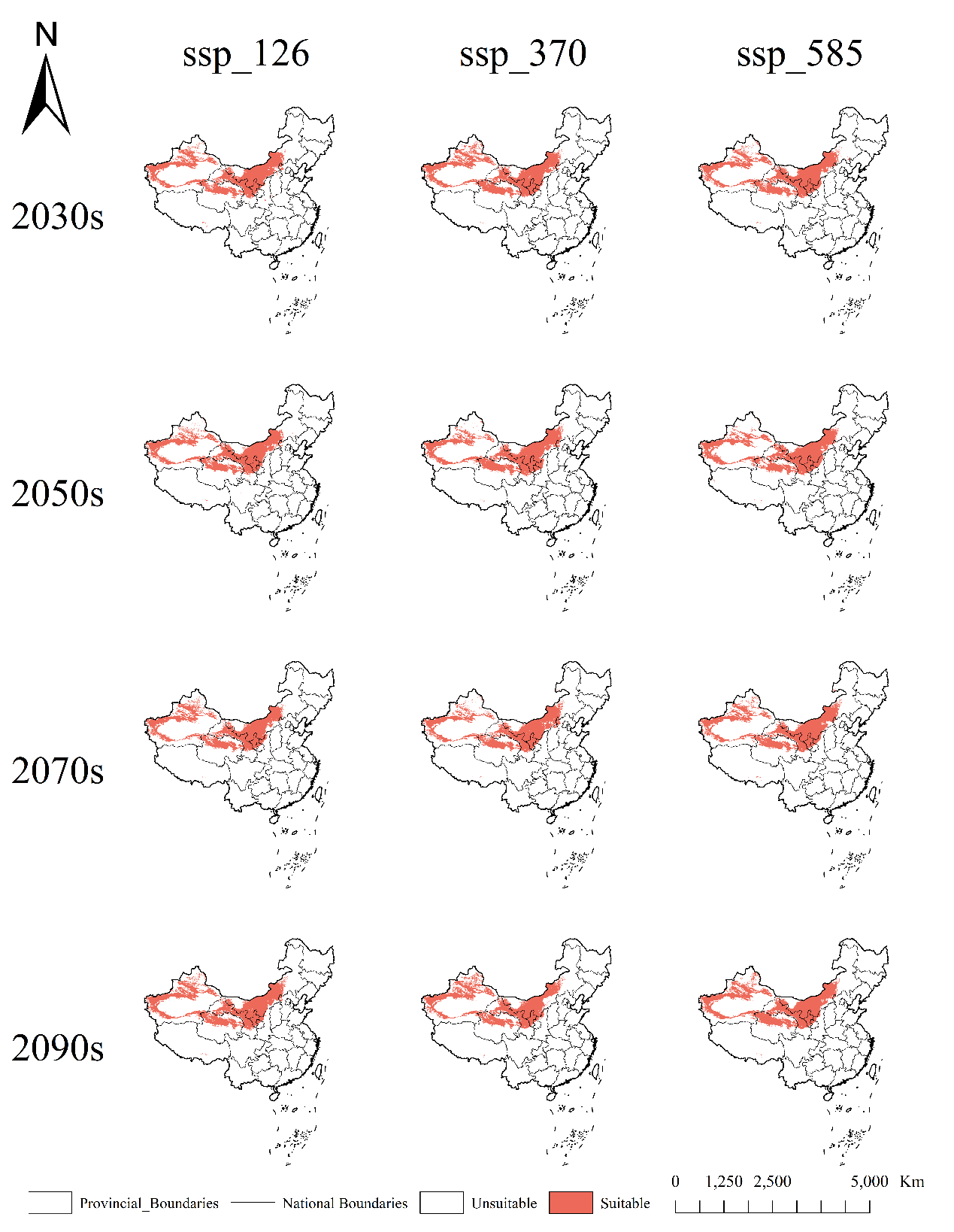


**Fig.S3 Binary map of the distribution of Reaumuria songarica in the future climate (Suitable/Unsuitable)**
